# Trophic transfer of carbon-14 from algae to zebrafish leads to its blending in biomolecules and the dysregulation of metabolism via isotope effect

**DOI:** 10.1093/nsr/nwae346

**Published:** 2024-09-30

**Authors:** Shipeng Dong, Renquan Deng, Hang Zeng, Pengfei Xue, Sijie Lin, Dongmei Zhou, Liang Mao

**Affiliations:** State Key Laboratory of Pollution Control and Resource Reuse, School of the Environment, Chemistry and Biomedicine Innovation Center, Nanjing University, Nanjing 210023, China; State Key Laboratory of Pollution Control and Resource Reuse, School of the Environment, Chemistry and Biomedicine Innovation Center, Nanjing University, Nanjing 210023, China; State Key Laboratory of Pollution Control and Resource Reuse, School of the Environment, Chemistry and Biomedicine Innovation Center, Nanjing University, Nanjing 210023, China; State Key Laboratory of Pollution Control and Resource Reuse, School of the Environment, Chemistry and Biomedicine Innovation Center, Nanjing University, Nanjing 210023, China; College of Environmental Science & Engineering, State Key Laboratory of Pollution Control and Resource Reuse, Tongji University, Shanghai 200092, China; State Key Laboratory of Pollution Control and Resource Reuse, School of the Environment, Chemistry and Biomedicine Innovation Center, Nanjing University, Nanjing 210023, China; State Key Laboratory of Pollution Control and Resource Reuse, School of the Environment, Chemistry and Biomedicine Innovation Center, Nanjing University, Nanjing 210023, China

**Keywords:** organically bound carbon-14, trophic transfer, isotope effects, metabolism dysregulation, isotopic substitution

## Abstract

Carbon-14 (C-14) has been a major contributor to the human radioactive exposure dose, as it is released into the environment from the nuclear industry in larger quantities compared to other radionuclides. This most abundant nuclide enters the biosphere as organically bound C-14 (OBC-14), posing a potential threat to public health. Yet, it remains unknown how this relatively low radiotoxic nuclide induces health risks via chemical effects, such as isotope effect. By establishing a trophic transfer model involving algae (*Scenedesmus obliquus*), daphnia (*Daphnia magna*) and zebrafish (*Danio rerio*), we demonstrate that rapid incorporation and transformation of inorganic C-14 by algae into OBC-14 facilitates the blending of C-14 into the biomolecules of zebrafish. We find that internalized C-14 is persistently retained in the brain of zebrafish, affecting DNA methylation and causing alterations in neuropathology. Global isotope tracing metabolomics with C-14 exposure further reveals the involvement of C-14 in various critical metabolic pathways, including one-carbon metabolism and nucleotide metabolism. We thus characterize the kinetic isotope effects for ^12^C/^14^C in the key reactions of these metabolic pathways through kinetic experiments and density functional theory computations, showing that the isotopic substitution of carbon in biochemicals regulates metabolism by disrupting reaction ratios via isotope effects. Our results suggest that inorganic C-14 discharged by the nuclear industry can be biotransformed into OBC-14 to impact metabolism via isotope effects, providing new insights into understanding the health risk of C-14, which is traditionally considered as a low radiotoxic nuclide.

## Teaser

Eating C-14 containing food impacts the metabolisms of fish by being blended in biomolecules, providing new insights on understanding the health risk of C-14.

## INTRODUCTION

With the rapid development of the nuclear industry in recent decades, concerns over the health risk associated with nuclides in the outflow from nuclear facilities are growing. Carbon-14 (C-14) is discharged into the environment by the nuclear industry in larger quantities compared to other radionuclides [1,2], with an annual global anthropogenic release of 3 × 10^14^ bq a^−1^ [3]. Releases of C-14 from nuclear reactors occur as operational gaseous or as liquid-borne emissions, entering the biosphere and posing health risks [4]. However, despite being the primary contributor to the collective radioactive dose in the global population, as estimated by the United Nations Scientific Committee on the Effects of Atomic Radiation (UNSCEAR) [5], C-14 remains one of the most understudied radionuclides [1]. In the aquatic environment, C-14 is primarily present in the form of dissolved inorganic carbon (DIC-14; dissolved ^14^CO_2_, H^14^CO_3_^−^ and ^14^CO_3_^2−^). Typical measured activity levels of C-14 ranged from 8.0 to 26.2 bq m^−3^, collected from seawater near the COGEMA La Hague nuclear plant (Goury) [6]. In groundwater near a nuclear reactor waste management area, the determined C-14 concentration ranged from 100 to 1800 bq L^−1^. This inorganic C-14 can be transferred to algae through photosynthesis as organically bound C-14 (OBC-14) in carbohydrates, lipids and proteins. The radioactivity of C-14 in typical algae (*Fucus serratus*) near the release site of a nuclear facility could be as high as 45.1 ± 0.5 Bq kg^−1^ wet weight, which is ∼3360 times higher than that of DIC-14 in water (Bq kg^−1^ ww/Bq L^−1^) [7]. For aqueous organisms inhabiting contaminated water, the uptake of C-14 via permeation of dissolved bicarbonate is quite limited. However, the radioactivity in these organisms can be much higher than that of DIC-14 in water [8], which is attributed to the direct ingestion and internalization of C-14 via predation of a producer with OBC-14. This trophic transfer of C-14 provides a pathway for the input of radiocarbon to animals and humans, threatening their health.

Although the effects induced by ionizing radiation in laboratory studies or public health are imperceptible [4], hypotheses regarding the genetic and somatic effects of C-14 via ionizing radiation have been raised for over 60 years [9]. Limited evidence supports the notion that C-14 is a low-radiotoxicity nuclide, estimated to have a dose factor 50 to 1000 times lower than that of ^137^Cs [10]. The toxicity induced by C-14-emitted ionizing radiation remains undetectable even under doses exceeding those found at nuclide-contaminated sites, given its status as a beta emitter with low penetrating ability. Unlike most radionuclides that accumulate in organisms as exogenous substances, C-14 is bioavailable to be internalized as a carbon unit participating in metabolism and life cycle, leading to long-term residue and severe risk [11]. Generally, isotopes are thought to possess similar physiochemical properties in metabolism, ensuring the parallel metabolic behavior of C-13/C-14 across different biomolecules relative to C-12 [12]. However, C-14 that is organically bound with biomolecules requires special attention, as the replacement of a carbon atom by a heavier C-14 isotope may disturb the binding affinities and reaction rates of critical biomolecules. Isotopically substituted molecules theoretically exhibit different reaction rates due to changes in bond orders and vibrational modes involving the replaced atoms of the reactant, known as isotope effects [13]. Kinetic isotope effects (KIEs), determined by the ratio of reaction kinetics of a molecule to that of an isotope-labeled molecule with heavier mass (*k*_light_/*k*_heavy_) [13], quantitatively illustrate the rate differences of isotopic molecules in the same chemical reaction. The maximal values of KIEs for ^12^C/^14^C and ^12^C/^13^C are calculated as 1.5 and 1.25 [14], respectively. This isotope effect is apparent in numerous aspects of molecular behavior, which is valuable for understanding the reactive site and interaction patterns of ligand–enzyme systems by evaluating binding isotope effects (BIEs) [15]. Substrates containing isotopes (e.g. ^12^C/^14^C) can exhibit differences in binding with enzymes, raising concerns about isotope-induced dysfunction of critical enzymes in metabolisms after incorporating C-14 via trophic transfer. The probability of metabolic dysfunction induced by isotope effects of substituted C-14 has not yet been reported.

One-carbon metabolism is constituted by the folate cycle and the methionine cycle. Protein serine hydroxymethyl transferase (SHMT) catalyzes the methylation of tetrahydrofolate (THF) to 5,10-methylene-tetrahydrofolate (me-THF), a key step in one-carbon metabolism, which has been linked to many diseases, including cancer [16]. Serine acts as the main carbon donor in this step by donating the carbon atom from its side chain to initiate the folate cycle, catalyzed by SHMT [17]. Methionine, through methionine adenyltransferase (MAT), is used to generate S-adenosylmethionine (SAM) to offer methyl groups for cellular methylation reactions, such as DNA methylation. Once the carbon of amino acid is substituted by C-14, a binding isotope effect is supposed to be observed on the catalytic reactions of SHMT and MAT, potentially disrupting one-carbon metabolism. Since one-carbon metabolism supports all cellular methylation reactions by providing major methyl sources of substrates [18], its dysfunction has been shown to influence DNA methylation and subsequent neuropathological alterations through the activation and repression of many genes [19,20].

Herein, we establish an aqueous food chain system using algae (*Scenedesmus obliquus*), daphnia (*Daphnia magna*) and zebrafish (*Danio rerio*), to simulate the trophic transfer of C-14 nuclide from producers to consumers through the transformation of inorganic soluble C-14 to OBC-14. By measuring the radioactivity distribution, we confirm the presence of C-14 in the brain of zebrafish as biomolecules. Global isotope tracing metabolomics with C-14 exposure is precisely characterized to reveal the participation of C-14 in various metabolic pathways, such as one-carbon metabolism. We also show the changes in the levels of DNA methylation and neuropathological alterations in fish to present the consequences of incorporating C-14. To elucidate the influence of C-14 substitution on enzyme reaction rates, we characterize the KIEs for ^12^C/^14^C in critical metabolic reactions. The results present the evidence that trophic transferred C-14 can organically bind with biomolecules, disrupting reaction ratios in critical metabolic steps via isotope effects, potentially inducing neuropathological and epigenetic alterations in organisms. This study provides new insights into understanding the health risks posed by C-14, a nuclide considered to have low radiotoxicity.

## RESULTS AND DISCUSSION

### Transformation of DIC-14 into OBC-14 by algae

**Figure 1.**
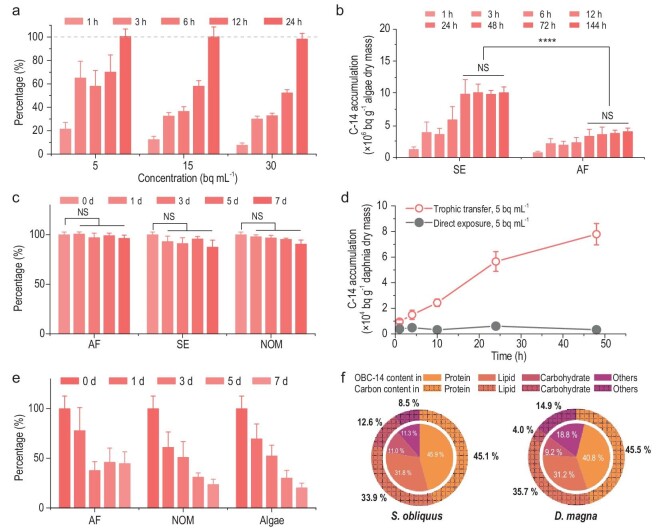
Transformation of inorganic C-14 to OBC-14 enabling accumulation and trophic transfer of C-14 in aquatic organisms. (a) Internalization percentage of dosed C-14 by algae. The gray dashed line represents the 100% of C-14 that was bio-utilized by algae. (b) Accumulation of C-14 in *S. obliquus* under cultivation in different medium containing NaHCO_3_ (AF) or not (SE) at a concentration of 15 bq mL^−1^. (c) The elimination of C-14 from algae under various culture media including AF, SE and AF containing 10 mg L^−1^ NOM. (d) Uptake of C-14 by *D. magna* through direct exposure to DIC-14 solution with a concentration of 5 bq mL^−1^ and trophic transfer from C-14-accumulated algae with a nominal concentration of 5 bq mL^−1^. (e) The elimination of C-14 from *D. magna* under various culture media including AF, NOM solution and AF containing 1 × 10^6^ cells/L algae. (f) Intrinsic percentage of carbon in carbohydrates, proteins and lipids of algae and daphnia (the outer circle) vs. the percentage of OBC-14 in carbohydrates, proteins and lipids in a corresponding organism after elimination (the inner circle). Data points are average values of three replicates; error bars indicate standard deviation. Asterisks (*) or NS indicate statistically significant (*P < 0.05, **P < 0.01, ***P < 0.001 and ****P < 0.0001) or insignificant differences.

To evaluate the transfer of C-14 from the aqueous system to biota, we first investigated the accumulation and transformation of C-14 in phytoplankton by culturing a typical freshwater algae *S. obliquus* in media containing various concentrations of ^14^C-labeled NaHCO_3_. No growth disturbance of algae was observed in the culture medium with the presence of radiocarbon during cultivation (Fig. S2). After 24 h of culture, all spiked C-14 (5, 15, 30, 350, 700, 1050 bq mL^−1^) in the selenite-enriched (SE) medium was transferred into algae (Fig. 1a and Fig. S3), with the radioactivity in the medium lower than the detection limit of ∼0.02 bq mL^−1^. In both SE medium and artificial freshwater (AF), spiked inorganic C-14 at a concentration of 15 bq mL^−1^ was rapidly absorbed by algae (Fig. 1b). The specific radioactivity of algae cultured in SE without DIC was measured as 9.84 × 10^3^ bq mg^−1^, higher than that of algae (3.29 × 10^3^ bq mg^−1^) cultured in DIC-containing AF. The calculated concentration factor (CF) was 6.7 × 10^5^ L kg^−1^ in SE or 2.6 × 10^5^ L kg^−1^ in AF, respectively, which are higher than the International Atomic Energy Agency (IAEA) recommended CF value for algae of 1 × 10^4^ L kg^−1^ [21]. The significant increase of C-14 in the growing community is attributed to the faster photosynthesis during growth, which converts DIC-14 into OBC-14 [22], facilitated by the carbon concentrating mechanism (CCM) employed by phytoplankton. Once OBC was formed and incorporated into algae via biosynthetic processes (e.g. photosynthesis), it was speculated to take longer to be eliminated [23]. Therefore, we performed the elimination of C-14 from algae by transferring exposed algae to radionuclide-free medium. We found that OBC-14 was hardly excreted from algae during a 7-day elimination process (Fig. 1c) in AF, SE or AF containing natural organic matter (NOM). Our results were consistent with a previous study in which accumulated C-14 was also hardly depurated by phytoplankton [24] and invertebrates [25] during the elimination period, suggesting the long-term retention of C-14 in algae.

To confirm the transformation of DIC-14 to OBC-14, we extracted the biomass components, including protein, lipid and carbohydrate, for radioactivity measurement and analyzed the relationship between the form of OBC-14 and the nutrient composition of algae (Fig. 1f). The radioactive OBC-14 in protein, lipid and carbohydrate accounted for 45.86% ± 1.90%, 31.84% ± 9.00% and 10.96% ± 0.76%, respectively, indicating that C-14 can be converted into biomass components through photosynthesis. The roughly consistent distribution of OBC-14 with the allocation of carbon in proteins (45.11%), lipids (33.85%) and carbohydrates (12.55%) in *S. obliquus* indicates that C-14 can be quickly absorbed, transformed and evenly blended in these components. The high bioavailability of C-14 in primary producers may enable the direct ingestion and internalization of C-14 by predators via trophic transfer, potentially leading to further input of radiocarbon to organisms at higher trophic levels and posing a threat to their health.

### Trophic transfer of OBC-14 in the food chain

To evaluate the contribution of trophic transfer of OBC-14 in the aquatic ecosystem, we selected *D. magna* and zebrafish (*D. rerio*) as consumers at higher trophic level to establish an aquatic food chain. The uptake of C-14 by daphnia via direct exposure to DIC-14 was compared to the digestion of OBC-14-accumulated algae. The C-14 body burden of daphnia shown in Fig. 1d indicates that the ingestion of OBC-14 is a much more efficient pathway for C-14 accumulation than direct exposure to DIC-14. Specifically, at 10 h, 24 h and 48 h, OBC-14 trophic transfer resulted in body burdens that were 7.6, 9.3 and 23.6 times higher than those resulting from direct exposure to DIC-14. It is well known that daphnia is a water filter organism, capable of retaining algae within its gut for digestion and utilization of organic carbon. Conversely, the HCO_3_^−^ ion should not be retained for utilization, resulting in the organic form of C-14 being more bioavailable than the inorganic form [24,26].

To evaluate the fixation of C-14 by daphnia via trophic transfer, we explored the elimination of C-14 in various depuration media. As shown in Fig. 1e, the radioactivity of C-14 gradually decreased over the test period, which was different from the minimal elimination observed in algae. More elimination of C-14 was observed in depurating medium (NOM or algae), which can reinforce the excretion of undigested food from the intestinal tract of daphnia, than AF, suggesting that the observed elimination is attributed to the undigested OBC-14-containing food. Once the food was digested, the incorporated OBC-14 remained within the predator, as demonstrated in the AF depuration medium (Fig. 1e). Notably, the calculated biomagnification factor (BMF) for the trophic transfer (see Text S2) was 0.03, significantly lower than 1.0 for the tested food chain, indicating the non-event of biomagnification in trophic transfer. Even so, our results indicate that organisms feeding on algae containing OBC-14 will accumulate high body burdens of C-14, highlighting the important role of the primary producer in introducing C-14 into the aquatic ecosystem. To confirm the incorporation of OBC-14 into the food via digestion, we further analyzed the distribution of OBC-14 in each nutrient component of daphnia (Fig. 1f). Following the ingestion of OBC-14 incorporated algae and subsequent elimination, the ingested C-14 was predominantly distributed in proteins (40.8% ± 2.3%), lipids (31.2% ± 2.6%) and carbohydrates (9.2% ± 1.1%), which aligns roughly with the intrinsic allocation proportion of carbon in the components of daphnia. However, due to the rather low proportion of carbohydrates in this planktonic crustacean [27], the OBC-14 in the carbohydrate of daphnia was much lower than that in algae. We assume that once OBC-14 is ingested by a predator, it follows the metabolic pathways of the organism(s) and distributes itself among the biochemical components accordingly.

### Trophic transfer induced C-14 retention in brains of fish

**Figure 2.**
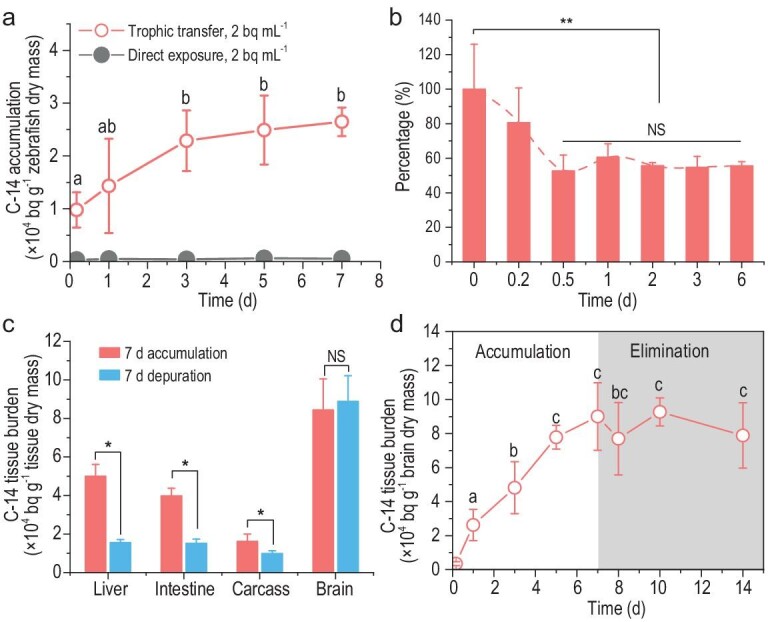
Trophic transfer of C-14 to zebrafish mainly being restrained in the brain. (a) Uptake of C-14 by *D. rerio* through direct exposure to DIC-14 solution with a concentration of 2 bq mL^−1^ or trophic transfer from C-14-accumulated *D. magna* with a nominal concentration of 2 bq mg^−1^. (b) The elimination of C-14 from zebrafish via continuous feeding of clean daphnia. (c) Distribution of C-14 in tissues of zebrafish after 7 days of accumulation and subsequently 7 days of depuration. (d) Uptake of C-14 in the brain of zebrafish during 7 days of exposure and subsequent 7 days of elimination. Data points with the same letter (e.g. a, b and c) are not significantly different from one another; asterisks (*) or NS indicate statistical significance (*P < 0.05, **P < 0.01, ***P < 0.001 and ****P < 0.0001).

Similar to daphnia, zebrafish can scarcely absorb C-14 when directly being exposed to DIC-14, reaching equilibrium at a C-14 body burden of 0.48 bq mg^−1^ on the first day. After 7 days of exposure, there was no notable accumulation of OBC-14 in the tissues of zebrafish, including the gill, intestinal tract, brain and carcass (below detection limit), indicating that DIC-14 was barely transferred and retained by the fish. While zebrafish were fed with OBC-14-containing daphnia, the C-14 body burden of the zebrafish increased at a rate of 4.56 bq mg^−1^ d^−1^ and reached a specific activity 52.6 times higher than the DIC-14 exposure group at 72 h (Fig. 2a), clearly demonstrating the efficient transfer of OBC-14 along the food chain. Biomagnification was not observed, as indicated by the calculated BMF value of 0.035, which is lower than 1.0. Despite this, the significant uptake of C-14 incorporated by prey as OBC-14 enhanced the bioavailability of C-14 to zebrafish, emphasizing the importance of trophic transfer in evaluating the internal exposure and ecological risk of C-14 to predators at higher trophic levels.

To elucidate the retention of C-14 in the tested fish, we monitored the elimination of C-14 from zebrafish that had accumulated OBC-14 via food chain exposure. The remaining fraction of accumulated C-14 in zebrafish (Fig. 2b) showed a decrease within 1 day, followed by a plateau with ∼60% of the total C-14 being retained in the fish with no further elimination. The accumulated C-14 via ingesting OBC-14-containing food partially excreted from the body due to the overdue digestion of food [28], similar to the trophic transfer from algae to daphnia. Once digestion is complete, the absorbed OBC-14 is utilized and incorporated into the fish's own tissues. In addition, we dissected C-14-accumulated zebrafish before and after 7 days of elimination to examine the changes in radioactivity across different tissues (Fig. 2c). After 7 days of OBC-14 delivery to the fish, the body burden of C-14 in the brain was measured as 84.52 ± 15.96 bq mg^−1^, representing the highest specific radioactivity among all tissues. The C-14 concentrations in the intestinal tract, liver and carcass were 39.87 ± 3.91, 50.03 ± 6.07 and 16.23 ± 3.77 bq mg^−1^, respectively. After 7 days of elimination, the C-14 content in the intestinal tract, liver and carcass decreased by 61.83%, 68.70% and 38.69%, respectively. However, the presence of C-14 in the brain remained at the same level, showing no significant change after 7 days of elimination. We monitored the enrichment and excretion dynamics of C-14 in the zebrafish brain, as the results in Fig. 2d show. During the initial 7 days of dietary exposure, ingested OBC-14 was continuously transferred to the brain at a rate of 12.52 bq mg^−1^ d^−1^, which is 2.74 times higher than the uptake rate for the whole zebrafish, suggesting the highly efficient fixation of OBC-14 in the brain. Notably, the high radioactivity in the zebrafish brain remained constant throughout the 7 days of duration period, indicating that the incorporated OBC-14 was retained as raw material for the synthesis of various biomolecules such as amino acids or genomic DNA [29], rather than being excreted. The integration of ^14^C into biomolecules ensures the *in vivo* maintenance of radionuclide for a long period, which could significantly participate in metabolism and construction of new neurons [30], potentially creating health risks.

### Blending of C-14 in the metabolism of biomolecules

Given the long-term retention of incorporated C-14 in the brain, we propose the blending of C-14 in biomolecules involved in the metabolism of biological molecules. In high-resolution mass spectrometry, ^13^C-labeled molecules will exhibit significantly higher ratio signals than the natural proportion at an isocharge-mass of M + 1, + 2, + 3 etc., according to its molecular formula or mass (e.g. C_3_H_7_NO_3_ could have 1, 2 or 3 carbons labeled) [31,32]. Similarly, in fish brain samples obtained through dietary exposure to OBC-14-containing food, signals at the isocharge-mass of M + 2, + 4 etc. should theoretically represent ^14^C-labeled molecules. Here, we used global isotope tracking metabolomics [33] for either ^13^C or ^14^C-labeled samples to identify labeled metabolites and the metabolic pathways they are involved in. UPLC-Qtof-MS/MS-based untargeted metabolomics were performed on both ^13^C or ^14^C-labeled samples, with unlabeled samples serving as control. Isotopically labeled metabolites were tracked, identified and annotated using the EL-MAVEN software (version 0.12.0) [34]. Peak detection was initially conducted across all samples by grouping peaks and screening peaks via scoring differences in retention time, intensities and any existing overlap between peaks. A standard spectral library was then used to annotate each peak for parent molecules or isotopologues. Finally, with annotated metabolites, a targeted list for isotopologues was generated and manually screened based on three main criteria: (i) good Gaussian shape of the peak; (ii) well grouped peaks with narrow retention time deviation; and (iii) relatively high intensities compared to the natural isotope baseline.

**Figure 3.**
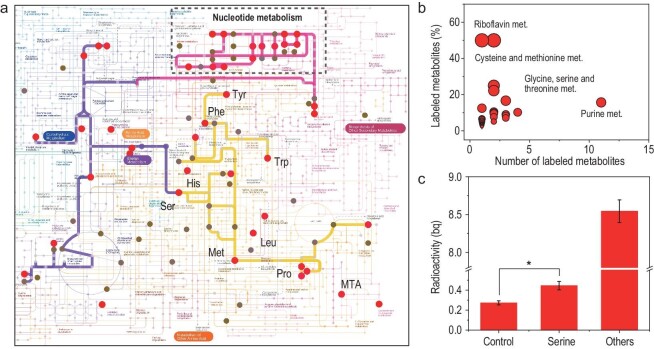
Blending of C-14 in the metabolism of biomolecules. (a) KEGG global pathway map of identified and annotated metabolites labeled in dietary exposed zebrafish brain by feeding with daphnia containing OBC-14 (red) or OBC-13 (brown). Representative ^14^C-labeled amino acids are marked as abbreviation. Inferred potentially active biosynthesis pathways are highlighted. Map generated using iPath tools (version 3) [39]. (b) Distributions of 47 ^14^C-labeled metabolites in zebrafish brain in the metabolic pathways. The circle size in the right panel represents the ratio of the number of labeled metabolites to the number of metabolites in a pathway. (c) Measured radioactivity in extracted serine by combined application of HPLC and LSC. In HPLC-LSC practice, a narrow section of eluent exactly at the retention time (RT) of serine in HPLC was flowed into LSC for in-time quantification on radiation signal. Asterisks (*) or NS indicate statistically significant or insignificant differences.

We thus obtained the global pathway map of identified and annotated OBC-14- or OBC-13-labeled metabolites in the zebrafish brain, as depicted in Fig. 3a. In the OBC-13-labeled sample, a total of 203 labeled metabolites from 52 metabolic pathways were tracked (Fig. S4 and Table S2). However, due to the extremely low mass of C-14 involved in the metabolism, suspected peaks close to the baseline of natural isotope abundance were discarded during the screening, resulting in the identification of only 46 OBC-14-labeled metabolites distributed across 37 metabolic pathways (Fig. 3b, Fig. S5 and Table S3). The results revealed a significant blending of C-14 into diverse areas of metabolism, including amino acids such as serine, methionine, histidine, tryptophan and tyrosine, which play vital roles in various metabolic processes. Interestingly, the distribution pattern of OBC-14-labeled metabolites showed an extensive blending of C-14 in a network of interrelated biochemical reactions involving one-carbon metabolism. In particular, the cysteine and methionine metabolism pathway displayed a relatively high labeling ratio, with serine, methionine, betaine and 5-methylthioadenosine (MTA) confirmed to be blended with C-14. In addition, the labeling of C-13 in threonine and 5-methyltetrahydrofolic acid (5-methyl-THF) also suggested the participation of a carbon isotope in methylation reactions (Table S3). As critical methyl donors or receptors, these biomolecules regulate the extent of methylation reactions (such as DNA methylation) in balance [16]. Notably, most labeling numbers and ratios of OBC-14-blended metabolites were distributed in purine metabolism or riboflavin metabolism, which were also deeply intertwined with one-carbon metabolism and DNA methylation. For example, one-carbon metabolism derived methyl transfer mediates the *de novo* purine biosynthesis [35], while in zebrafish, disruption of purine metabolism was observed once DNA methylation was dysregulated [36]. In riboflavin metabolism, riboflavin (FAD) serves as one of the sources of coenzymes which participate in one-carbon metabolism, modulating DNA methylation via reprogramming energy metabolism or by dysregulating the enzyme activity [37,38]. On the back of evidence that C-14 incorporation is highly related to one-carbon metabolism, we further quantified the radioactivity of C-14 in brain extracted serine, a vital donor for *de novo* biosynthesis of carbon units. By performing in-time liquid scintillation counting (LSC) quantification on the narrow section of the eluent precisely at the retention time of serine in high-performance liquid chromatography (HPLC), significantly higher radioactivity was detected in serine extracts compared to control samples where no radioactive signals were present (Fig. 3c). Analytical standards of L-serine and ^14^C-serine were simultaneously measured to ensure reliable HPLC-LSC performance (Fig. S6). Aside from its involvement in one-carbon metabolism via blending in amino acids, the integration of C-14 into the DNA backbone warrants significant attention, given the observation that C-14 is assimilated into key biomolecules involved in nucleotide metabolism, such as guanosine, uridine, uracil and their derivatives (Fig. 3a and Table S3). The presence of C-14 in these fundamental molecules underscores its potential impact on the stability and function of genetic material. In the ^13^C-labeled metabolites, more nucleotides, including adenine, guanine, thymidine and uracil, were blended with the isotope, further indicating the integration of C-14 into the backbone of DNA. Collectively, our findings demonstrate that trophic transfer of OBC-14 to zebrafish leads to the blending of C-14 into biomolecules in the brain and participation in various critical metabolic pathways, including one-carbon metabolism and nucleotide metabolism.

### Health risks of incorporated OBC-14

**Figure 4.**
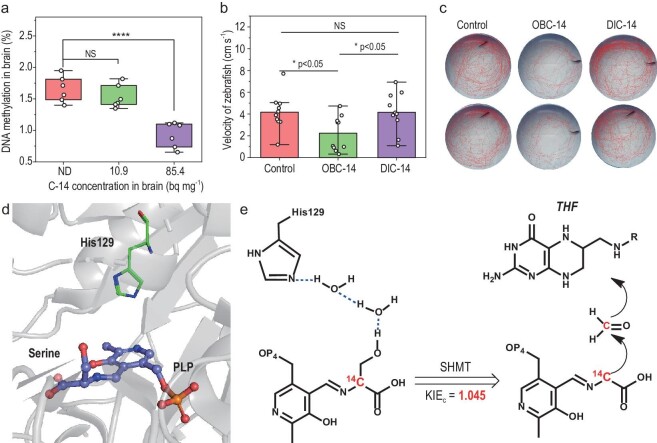
Significant effects associated with one-carbon metabolism induced by incorporated C-14 in zebrafish. (a) Alteration of global DNA 5mC methylation level after accumulating no (not detectable, ND), low (10.9 bq mg^−1^) and high (85.4 bq mg^−1^) concentrations of C-14 in zebrafish brain. (b) Average velocity and corresponding (c) representative trajectories recorded from zebrafish ingested OBC-14 via trophic transfer (with a fish body burden of 13.9 bq mg^−1^) or directly exposed to DIC-14 medium at a higher concentration of 1000 bq mL^−1^. (d) A prediction of the geometry showed that serine substrate combined with PLP aldimine in the SHMT structure (PDB ID codes 4OT8) approached to the His129. (e) KIE_c_ predicted for the rate-limiting TS as His129-PLP-serine composite for the donation of a methyl to THF. (*) indicates statistically significant differences (*P < 0.05, **P < 0.01, ***P < 0.001 and ****P < 0.0001).

Exposure to radionuclides such as H-3 has been shown to induce DNA damage once integrated into fish. However, evidence regarding the health risks associated with C-14 is as yet rather limited, due to the inorganic carbon exposure method that being applied in the evaluation. In current trophic transfer exposure, we observed a significant decrease in DNA methylation (5mC) levels in the fish brain, as shown in Fig. 4a, along with a slight decrease in RNA methylation (m6A, Fig. S7). At a low dose of C-14, retained in the fish brain (10.9 bq mg^−1^), no significant difference in degree of DNA methylation in the zebrafish brain was observed compared to the control group. However, when the dose of accumulated C-14 in the brain increased to 85.4 bq mg^−1^, the degree of DNA methylation decreased by 43.95% compared to the control group. Alterations in DNA/RNA methylation have been proven to be related to the inhibition of the upstream reactions of the methyl donors such as serine and methionine in one-carbon metabolism [16,40]. Given that trophic transfer exposure delivers C-14 blending in these critical metabolites (Fig. 3), we propose that the blending of C-14 in the metabolism of the brain may create an imbalance among critical protein components, such as amyloid beta and amyloid precursor protein, leading to neurological and behavioral abnormalities [41]. We thus assessed the behavioral alterations in zebrafish after incorporating C-14 into their brains. No lethality was observed in the visual model for studying behavioral pathology. However, a reduction in locomotion was noted in fish that accumulated C-14 compared to unexposed fish, as recorded by the trajectory of each fish (Fig. 4b and Fig. S8). Significant reductions in both average velocity and total traveled distance (Fig. 4c and Fig. S9) were observed in fish that ingested OBC-14 via trophic transfer rather than those directly exposed to DIC-14 medium at a higher concentration (*P* < 0.05), due to the difference in the accumulated dose of C-14. Since the alleviated mobility was proven to be related to the disorders of neuro-related genes in the zebrafish brain [42,43], we further selected two neuro-related genes, *dmbx1a* and *otx2b*, as verification indicators to explore the possible influence on zebrafish exercise ability by evaluating their relative RNA expression. These two genes are strongly expressed in the diencephalic and midbrain regions, which are essential for regulating the morphology and function of the central nervous system [44]. After incorporating C-14 into the fish brain, the relative expression of *dmbx1a* and *otx2b* was significantly downregulated by 72.47% and 65.70%, respectively, while direct exposure to DIC-14 did not significantly impact gene expression (Fig. S10), consistent with the observed suppression of locomotion under different exposure pathways. As a major epigenetic factor, DNA methylation determines gene expression alongside other regulatory proteins [45]. Alterations in the DNA methylation levels of genes such as *dmbx1a* and *otx2b* can depress the corresponding expression, which further regulates neurogenesis in the ventral tegmental area (VTA) and causes dysfunction in the central nervous system [46]. Therefore, disturbances in DNA methylation in the fish brain may lead to impaired neurodevelopment in juveniles or behavioral abnormalities in adults, [47] as the current work observed. Given the crucial role of DNA methylation in brain development, alterations at the promoters of genes, such as *Slit1, Bdnf, Wnt3, Esrrb* and *Tcl1*, may also result in transcriptional changes and influence the neural behavior of organisms [48]. In summary, owing to the blending of C-14 in metabolites that participate in metabolism, the DNA methylation level is altered in the fish brain to cause neurotoxic effects.

The involvement of C-14 in metabolism needs specific attention because the substitution of C-12 with the heavier C-14 isotope may disturb the binding affinities and reaction rates of critical biomolecules. Isotopically substituted molecules theoretically exhibit different reaction rates due to changes in bond orders and vibrational modes of the replaced atoms, known as isotope effects [13]. In the current work, as the primary carbon donor in one-carbon metabolism for DNA methylation, serine has been identified as participating in the metabolism of the zebrafish brain with C-14 incorporation (Fig. 3). We performed a density functional theory (DFT) calculation (6–31G∗ basis set) [49] to evaluate the vibrational frequency shift of serine induced by C-14 substitution at the carbon atom where enzymatic cleavage of the C–C bond produces a carbon unit for further methylation reaction. The data showed significant downshifted vibrational peaks in the spectrum of serine (Fig. S11), such as the specific assignment of *γ*CH_2_ at 1000 cm^−1^ to 984 cm^−1^, δC-OH at 1271 cm^−1^ to 1248 cm^−1^ and νC_α_–C at 832 cm^−1^ to 831 cm^−1^ [50]. Owing to the altered vibrational frequencies caused by isotopic substitution, the catalytic reaction of serine mediated by SHMT was expected to be regulated. We thus conducted the frequency calculations of ^14^C-serine (^14^C substitution at position 3 in serine where demethylation occurs) to calculate theoretical kinetic isotope effects (^12^C/^14^C, KIE_C_) with the ISOEFF package [51]. In SHMT, serine is first covalently linked to pyridoxal 5′-phosphate (PLP), forming a PLP–serine aldimine structure that is involved in the transfer of carbon [52]. Priorly performed geometry optimizations and frequency calculations suggest that the residue His129 at the proximal end of the SHMT conformation accepts protons from the serine hydroxyl functional group (Fig. 4d). Two water molecules at the reactive site act as a bridge to participate in the cleavage of the C−C bond (2.13 Å), promoting the transfer of protons. The process of forming aldehyde intermediates via cleavage of the C−C bond accounts for the rate-determining step (37.6 kcal/mol), which is a strong endothermic process. The intrinsic KIE for the cleavage of the C−C bond of serine was calculated from the rate-limiting transition states (TS) as His129-PLP-serine composite, yielding a final KIE_C_ (C3-^14^C) value of 1.045 (Fig. 4e), indicating a possible alleviation in this reaction. This donation of a methyl to THF initiates one-carbon metabolism [16,17]. A profound KIE (Me-^14^C) value of 1.11 has been observed in methyl-transfer TS of C-14-substituted SAM, which is also a critical methyl donor. This high KIE value of 1.11 is consistent with a previously reported calculated KIE result of 1.106 for the Human DNMT1 TS2 and experimental result of 1.107 [53]. In the current work, C-14 substitution was found in many amino acids participating in critical metabolic pathways, as well as other metabolites, raising concerns about the isotope-effect-induced dysfunction of critical enzymes in metabolisms following the incorporation of C-14 via trophic transfer.

## MATERIALS AND METHODS

### Test organisms

All tested organisms, including *S. obliquus, D. magna* and *D. rerio*, were obtained from the Institute of Hydrobiology, Chinese Academy of Sciences (Wuhan, China). Algae (*S. obliquus)* was propagated in SE medium for more than 7 days to obtain a sufficient population. Newly born daphnia (*D. magna*) was cultured and acclimated in aerated AF for 3 days by feeding once every two days with a culture of algae before use. The above culture conditions were set at 25 ± 1°C, with a 14 h light : 10 h dark photoperiod. The AB wild-type zebrafish (*D. rerio*) were maintained at 28 ± 0.5°C with a 14 h light : 10 h dark cycle. Zebrafish were cultured and acclimated in aerated water for more than 7 days by daily feeding with 0.3 mg daphnia (dry weight, 15–20 individuals) per fish, which is equal to ∼1% of the dry weight of a fish. Feeding was stopped 1 day before initiating experiments, with animals taken at random from a stock tank. Algae, daphnia neonates (∼3 days old) and growing zebrafish (∼1 month old) were used for following uptake, depuration and trophic transfer experiments.

### Accumulation of C-14 in algae, daphnia and zebrafish

Accumulation of C-14 in algae was performed by exposing the organisms to DIC-14-containing medium. *D. magna* was exposed to C-14 through two pathways: direct exposure to DIC-14-containing medium or trophic transfer by feeding C-14-accumulated algae. For zebrafish accumulation and distribution investigation, OBC-14-accumulated daphnia was obtained by feeding them with OBC-14-accumulated algae (text S3). Subsequently, zebrafish were exposed to either DIC-14-containing medium or via trophic transfer by feeding C-14-accumulated daphnia. To quantify the C-14 in various organs of zebrafish, C-14-accumulated zebrafish before and after 7 days of elimination were sacrificed and preprocessed as described above. Their brain, gill, gut and carcass tissues were harvested, freeze-dried, weighed and the radioactivity was determined, with detailed radioactivity quantification methods described in text S4. A detailed description of the accumulation of C-14 in algae, daphnia and zebrafish is presented in text S5.

### Determination of C-14 in biochemical components of organisms

After exposure for 7 days in medium containing DIC-14, followed by a 3-day elimination period in C-14-free medium, the algae cells were collected and washed at least three times. To measure the radioactivity of C-14 in each biochemical component of the organisms, sequential extractions of lipid, protein and carbohydrate components were performed to avoid possible contamination and mass loss [54]. A 0.4 g sample of algae cells was placed in a 10-mL centrifuge tube and then treated with 6 mL of methanol. The mixture was incubated at 70°C for 1 h, followed by sonication for additional 30 min. The mixture was then centrifuged at 5000 r/min for 2 min. The supernatant and sediment were separately collected for further treatment. The sediment was washed with 6 mL of methanol twice more. All the supernatants were collected, mixed and subjected to methanol evaporation to obtain the lipid content [55], which was subsequently measured for radioactivity by LSC. To obtain the protein content, the pellets were processed using the acetone precipitation method [56], which was then dissolved in 5 mL of 3% KOH solution and incubated at 80°C for 1 h. After sonication for 30 min, the mixture was mixed with 2 mL chloroform and *n*-butyl alcohol solution (v: v = 4 : 1) for 10 min of shaking, followed by centrifugation at 5000 r/min for 2 min. The extraction process was repeated at least five times to ensure complete isolation of the protein content. The radioactivity in the obtained protein content was determined by LSC. For carbohydrate separation [57], algae cell pellets were extracted using the methanol method described above. After obtaining the sediment via centrifugation, 10 mL of ethanol was added in it, followed by thoroughly mixing and centrifugation for precipitation collection. The process was repeated three times. All collected precipitation was mixed with a scintillation cocktail and measured by LSC for quantifying the radioactivity. The intrinsic allocation of carbon in each biochemical component of algae was determined using methods described in text S6. The extraction and measurement of C-14 in biochemical components of *D. magna* were similar to that of algae. Since the directly exposed daphnia can barely transform DIC-14 to OBC-14, the C-14 allocation in biochemical components of daphnia was investigated only for the trophic transfer treatment.

### Identification of C-14 blending in metabolites of algae and zebrafish brain

OBC-14-accumulated algae and daphnia were prepared as described above, at the highest exposure concentration of 1050 bq mL^−1^. After 3 days daily feeding of C-14-accumulated daphnia, the exposed zebrafish via trophic transfer were transferred to the elimination scenario for 3 days. The C-14-accumulated zebrafish were sacrificed to obtain brain tissues via dissection. After dissection, the brain samples were quickly frozen using liquid nitrogen, and stored at −80°C. To enhance the detected signals, ten brain samples were mixed as one replicate. For metabolite extraction, algae or brain samples were homogenized with 200 μL of H_2_O and 20 ceramic beads (0.1 mm) using the homogenizer. Samples were extracted with 800 μL of MeOH, followed by 10 min of sonication (50 Hz) at 4°C. To precipitate protein, samples were mixed with 1 mL of 6 M HCl solution for 2 h of incubation, followed by 15 min centrifugation using 16 200 × g to remove the insoluble material. Supernatants were transferred to HPLC glass vials and stored at −20°C prior to LC/MS analysis or combined application of HPLC and LSC. Control samples were obtained by culturing the zebrafish fed by daphnia that did not accumulate C-14, with the same dissection and metabolite extraction operation. Detailed parameters for HPLC and MS analysis are presented in text S7.

### Effects of C-14 on zebrafish

*Behavior disturbance*. Thirty zebrafish of similar age (∼1 month old) were divided into three groups for control, direct exposure and trophic transfer exposure treatment and solely cultured in a container. Compared to the normally fed control group, the direct exposure medium was spiked with DIC-14 to an activity concentration of 50 bq mL^−1^, and the diet of the trophic transfer exposure group was replaced with the equal mass of OBC-14-containing daphnia at a body burden of 50 bq mg^−1^. After 7 days of treatment, each zebrafish was placed in a circular glass dish filled with 1 L of aerated water, with vertical recording of the movement trajectory of each for 10 min by Noldus Ethovision® XT16 (Noldus Information Technologies, Inc.) tracking software. The first minute was considered an acclimatization period, thus excluded before analysis.

*DNA methylation.* After being exposed to C-14 via daily feeding of OBC-14-containing daphnia at a body burden of 100 or 750 bq mg^−1^ for 7 days, the zebrafish were dissected to obtain brain tissue. Zebrafish that were fed with C-14-free daphnia were exposed as the control. The body burdens in the brain of exposed zebrafish were measured as 10.93 or 85.40 bq mg^−1^ respectively, for either feeding concentration. The obtained brain was immediately homogenized for the extraction of DNA using a DNeasy Tissue Kit (Qiagen). Global DNA methylation quantification was spectrophotometrically performed by measuring the absorbance at 450 nm using an Imprint Methylated DNA Quantification Kit (Merck).

### Statistics

All statistical analyses were performed using SPSS 18.0 (PASW Statistics, IBM Company); differences were considered statistically significant at *P* < 0.05. Depuration results were analyzed by one-way analysis of variance (ANOVA). Errors always represent standard deviation values.

## Supplementary Material

nwae346_Supplemental_File
