# Supplementary material for: Trophic transfer of carbon-14 from algae to zebrafish leads to its blending in biomolecules and the dysregulation of metabolism via isotope effect
Source: Natl Sci Rev. 2024 Sep 30;12(1):nwae346. doi: 10.1093/nsr/nwae346 (PMC11706001; doi:10.1093/nsr/nwae346)
Supplement: nwae346_Supplemental_File [file nwae346_supplemental_file.pdf]

Supplementary Information

Trophic transfer of carbon-14 from algae to zebrafish lead its blending in biomolecules and dysregulating metabolism via isotope effect

Shipeng Dong, Renquan Deng, Hang Zeng, Pengfei Xue, Sijie Lin, Dongmei Zhou and Liang Mao*

*Corresponding author. Email: lmao@nju.edu.cn.

This PDF file includes:

Supporting Text

Figures. S1 to S11

Table S1 to S3

Materials and Methods

S1. Chemicals

All reagents used are of analytical grade without further purification. The carbon-14 labeled sodium bicarbonate was purchased from China Isotope & Radiation Cor. Beijing). Artificial freshwater (AF) and selenite enrichment medium (SE) were prepared according to ingredients and adjusted to pH 8.3. All used reagents, including sodium nitrate ($NaNO_3$), dipotassium hydrogen phosphate trihydrate ($K_2HPO_4 \cdot 3H_2O$), magnesium sulfate heptahydrate ($MgSO_4 \cdot 7H_2O$), calcium chloride dihydrate ($CaCl_2 \cdot 2H_2O$), potassium dihydrogen phosphate(KH_2PO_4), sodium chloride (NaCl), ferric chloride hexahydrate ($FeCl_3 \cdot 6H_2O$), ethylenediamine tetraacetic acid disodium salt (Na_2EDTA), manganese chloride tetrahydrate ($MnCl_2 \cdot 4H_2O$), boric acid (H_3BO_3), zinc sulfate heptahydrate ($ZnSO_4 \cdot 7H2O$), copper sulfate pentahydrate ($CuSO_4 \cdot 5H_2O$), ammonium heptamolybdate ($(NH_4)6Mo_7O_{24}$), and hydrochloric acid (HCl, 37%), were purchased from Nanjing Chemical Reagent Co., Ltd. Natural organic matter (NOM) was obtained from the International Humic Substances Society.

S2. Concentration factor (CF) and Biomagnification factor (BMF) calculation

Concentration factor (CF) refers to the ratio of the concentration of a substance in the organism to the concentration in the surrounding environment, which indicates how much the concentration of substances has been concentrated in the steady-state. To obtain the concentration factor (CF) for tested aquatic organisms that absorbed C-14 from direct exposure to DIC-14 medium, a one-compartment kinetic model was applied and fitted by the uptake concentrations in time course.[1] The model was suit for current exposure period under nonsteady-state conditions, which is described as follows:

$$\frac{dC}{dt} = k_{in}C_w - k_{out}C \qquad \text{Eq1}$$

where C is the instant C-14 concentration in tested organism (mg/g); t is the exposure time (h^{-1}); k_{in} is the influx rate constant ($L\ Bq^{-1}\ h^{-1}$), described the total uptake of C-14 to the corresponding concentration in medium; C_w is the C-14 concentration in medium (Bq/L) and assumes to be constant, and k_{out} is the efflux rate constant (h^{-1}), reflected the elimination rate of accumulated C-14 from the organisms. Under the condition where equilibrium state reached, the body burden at steady state $C_{ss} = k_{in}C_w/k_{out}$, CF_k can be

calculated by:

$$\mathrm{CF}_k = k_{in}/k_{out} \qquad \text{Eq 2}$$

Thus, CF value could be calculated as the ratio of influx rate constant and efflux rate constant via first order kinetics fitted model. Using this model, CF was obtained for tested organisms. For the condition where elimination rate was not measurable, CF was obtained by calculating the ratio of C-14 concentration in tested organism at equilibrium to medium C-14 concentration.

Biological magnification factor (BMF) is a term used in ecotoxicology to quantify the increase in concentration of a persistent, non-biodegradable substance as it moves up the food chain from one trophic level to another. The BMF is a crucial concept in environmental risk assessment and management, particularly for ecosystems where there are long food chains and concerns about human consumption of contaminated species. To calculate the biomagnification factor (BMF) in trophic transfer studies, the assimilation efficiency (α) and elimination rate (k_{out}) is needed to priorly obtain. [2] The chemical assimilation efficiency is calculated as:

$$\alpha = \frac{C_{0,d} \cdot k_{out}}{I \cdot C_{food}} \cdot \frac{1}{1 - e^{-k_{out} t}} \qquad \text{Eq 3}$$

where $C_{0,d}$ is the C-14 concentration in predator at time zero of the depuration phase (mg/g); k_{out} is the efflux rate constant (h^{-1}); concentration in food (mg g^{-1} food); I is food ingestion rate constant (mg food mg^{-1} predator day^{-1}). BMF can be calculated by multiplying α with the feeding rate constant I and dividing the product by the overall depuration rate constant k_{out}:

$$BMF = \frac{I \cdot \alpha}{k_{out}} \qquad \text{Eq 4}$$

put α into Eq 4, the BMF can be calculated as:

$$BMF = \frac{C_{0,d}}{C_{food}} \cdot \frac{1}{1 - e^{-k_{out} t}} \qquad \text{Eq 5}$$

S3. Preparation of C-14 accumulated daphnia as food for zebrafish

Similarly, to prepared the predetermined C-14 accumulated daphnia used as the food of zebrafish, we

amplified the exposure dose of DIC-14 and the culture volume. Briefly, a 5-L beaker containing 4 L of culture media was prepared with one thousand daphnia neonates, where were fed with 100 Bq g^{-1} C-14 accumulated algae. The daphnia was cultured in an incubator, and the temperature was kept at 26-28 ℃ under continuous light illumination. After culturing for 3 d, the daphnia were washed with clean culture medium for at least three times and transferred to C-14 free medium for another 3 d elimination. The daphnia were then harvested, washed and subjected to the determination of radioactivity.

S4. Radioactivity Quantification

The radioactivity of the ^{14}C incorporated samples were quantitatively measured by liquid scintillation counter (LSC; Tri-carb 5110TR, PerkinElmer) following combustion in a biological oxidizer (BO; Pyrolyser-6 Trio). The radioactivity of liquid samples was directly added to scintillation cocktail (Gold Star, Meridian) for LSC quantification. The BO was used to oxidize the ^{14}C incorporated samples at 680 °C for 10 min under a stream of oxygen gas running at 360 mL/min. The $^{14}CO_2$ released during the combustion process was captured in alkaline ^{14}C scintillation cocktail (Zinsser Analytic, Germany) and then counted by LSC. BO has a high recovery rate (> 99%) for collecting both DIC-14 and OBC-14.

S5. Accumulation of C-14 in algae, daphnia and zebrafish

Detail description on the accumulation of C-14 in algae, daphnia and zebrafish was presented in Supplementary text SPrior to the exposure, algae were washed three times with AF and concentrated to approximately 10^8 cells mL^{-1} by centrifugation at 3000rpm. This algal suspension was added to 100 mL of AF or selenite Enrichment (SE) medium (in a concentration of 10^6 cells mL^{-1}) containing ^{14}C-labeled sodium bicarbonate at various concentrations of 5, 15, 30, 350, 700, 1050 bq mL^{-1}. Triplicate control (without algae) containers with 100 mL of exposure medium were prepared to quantify the loss of ^{14}C during the exposure period as well. The radioactivity of medium in each container was obtained by mixing 1 mL of medium with 2 mL of Gold Star scintillation cocktail for LSC measurement. After exposure for 1, 3, 6, 12, 24, 48, 72 and 144 h, algae were washed three times with AF to remove free C-14 attached to the algae surface and sampled by centrifugation. The cell number of each sample were counted before radioactivity quantification. To discriminate the measurement of DIC-14 or OBC-14 in algae, we measured the radioactivity of homogenized algae samples that being washed by 0.1 mM HCl to remove DIC or not. The result showed no

significant difference between the samples (Supplementary Fig. 1), indicating the DIC-14 in algae was negligible compared with OBC-14. The C-14 mass balance and elimination results (Fig. 1) also support the complete transformation of DIC-14 to OBC-14 by algae. Thus, the radioactivity of the C-14 in algae was directly measured by LSC after homogenization. No fading algae or decreasing of cell numbers were observed during the exposure period. Algae samples were ultrasonicated for 30 min and added to scintillation vials with 15 mL of Gold Star cocktail for LSC measurement. The algae that have been exposed in DIC-14 containing SE medium for 144 h was washed 3 times and transferred to containers with various C-14 free elimination media including AF, SE and AF containing 10 mg L^{-1} natural organic matter (NOM). At each predetermined sampling interval, the radioactivity of medium and algae were determined using above mentioned LSC measurements.

D. magna were exposed to C-14 through two pathways: Direct exposure to DIC-14 containing medium or trophic transfer by feeding C-14 accumulated algae. When being directly exposed to DIC-14, *D. magna* (~ 3 day old) were exposed to DIC-14 containing AF at a concentration of 5 bq mL^{-1} for 48 h without agitation. At each sampling time interval, 10 individuals were collected as one sample and the C-14 uptake by the daphnia was quantified. To confirm the chemical form of measured C-14, the daphnia was homogenized and washed with 0.1 mM HCl to remove DIC. The radioactivity was measured lower than detection limit. Thus, we assume the measured C-14 in daphnia via direct exposure should be DIC-14. In the trophic transfer of C-14 from algae to daphnia, Daphnia were cultured with OBC-14 accumulated algae (nominal concentration of medium at 5 bq mL^{-1}) to quantify the uptake of OBC-14 when algae were the only food source. OBC-14 accumulated algae were obtained by preculturing algae in C-14 containing SE medium for 24 h and centrifugation. The algae pellet was resuspended and the nominal C-14 concentration dose supplied to daphnia was 5 bq mL^{-1}. At each sampling time interval, all the daphnia from triplicate vials were transferred to clean medium for heavily rinsing to remove the attached algae on the surface, followed by the C-14 quantification. The daphnia that have been accumulated C-14 via directly exposure or trophic transfer was rinsed and transferred to containers with various C-14 free elimination media including AF, NOM solution and AF containing 1×10^6 cells/L algae. At each predetermined sampling interval, the radioactivity of medium and daphnia were determined.

For zebrafish accumulation and distribution investigation, the zebrafish has been acclimatized for at least one week by feeding daphnia. No abnormal behavior or death was observed during the uptake and depuration experiments. Each adult zebrafish was put into a beaker containing 150 mL of test medium.

OBC-14 accumulated daphnia was obtained by feeding them with OBC-14 accumulated algae as described above. In trophic transfer exposure scenario, zebrafish was cultured in C-14 free medium and fed with C-14 accumulated daphnia (20 individuals with a body burden of 750 bq/mg, roughly equal to 1% of dry mass of single zebrafish) daily for 7 days. The preparation of C-14 accumulated daphnia was described in Supplementary text S4. For the direct exposure to DIC-14, zebrafish was exposed to medium containing 2 bq mL^{-1} of ^{14}C-labeled sodium bicarbonate with daily feeding of 20 daphnia individuals as the only food. Daphnia were observed to be rapidly consumed within 5 min. After 1 h feeding, the culture media renewal was applied daily. At each predetermined interval, zebrafish was euthanized using 3-Aminobenzoic acid ethyl ester methanesulfonate (MS-222, 0.015%), the body length was measured, and then epidermis was peeled and fish fins was cut to exclude the C-14 adhering to the surface. Subsequently, medaka were freeze-dried, weighed and the radioactivity was determined. The radioactivity of control samples (i.e., zebrafish without radioactive exposure) was subtracted from the uptake results. Elimination experiments were conducted after the C-14 uptake by zebrafish via either direct exposure or trophic transfer. C-14 accumulated zebrafish was transferred to C-14 free medium and fed with 20 daphnia once a day for 6 days. At each predetermined interval, the quantification of C-14 in zebrafish was performed as described above. To quantify the C-14 in various organs of zebrafish, C-14 accumulated zebrafish before and after 7d elimination were sacrificed and preprocessed as described above, then their brain, gill, gut and carcass tissues were harvested, freeze-dried, weighed and the radioactivity was determined as described above.

S6. Intrinsic allocation of carbon in each biochemical component of algae and daphnia determination

The chemical composition of *S. obliquus* biomass was characterized according to lipid, protein, carbohydrate content. Algae suspension was centrifuged at 5000 rpm for 5 min to obtain algae pellet, which was then mixed with 10 mL of 0.5 $mol \cdot L^{-1}$ NaOH solution for following 5 min incubation in boiled water bath. The mixture was cooled and centrifuged again at 5000 rpm for 5min. The supernatant was pipetted and transferred to a centrifuge tube for another mixing with 5 mL of 0.5 $mol \cdot L^{-1}$ NaOH. Repeat the above operation and mix the two extracts as the sample to be tested.

To determine the protein content[3], a batch of standard protein solutions with various concentrations (25, 50, 100, 200, 300, 400, 500 ug/mL) were priorly prepared using bovine serum blood protein (BSA). Protein concentration in samples were determined by adding 1 mL of sample and a gradient concentration of protein standard solution to 5 mL of 0.1 $g \cdot L^{-1}$ Coomassie Brilliant Blue solution (50 mL of 95% ethanol +

100 mL of 85% phosphoric acid priorly was prepared) for absorbance measurement at 595 nm after full mixing.

To determine the carbohydrate content, a classic phenol-sulfuric acid method was applied with modification[4]. After extraction procedure using the method described above, 5 ml of 0.5 mol/L H_2SO_4 was added into it and diluted to 25 ml. Then a volume of 0.5 ml extracted solution was added to 1 ml of 6% phenol and 5 ml of 98% concentrated sulfuric acid, and then leave it at room temperature for 0.5h after shaking. A batch of standard glucose solutions with various concentrations (0.1, 0.2, 0.3, 0.4, 0.5, 0.6 mg/mL) were priorly prepared. Carbohydrate concentration in samples were determined by adding 2 mL of sample and a gradient concentration of glucose standard solution to 6 mL of 1 $g \cdot L^{-1}$ anthrone solution (prepared with 80% concentrated sulfuric acid)[5]. After thoroughly mixing by shaking and incubated in boiled water bath for 15 min, the absorbance of the mixture was measured at 490 nm after quick cooling to room temperature.

To determine the lipid content, a batch of standard lipid solutions with various concentrations (0.5, 1.0, 2.0, 4.0, 6.0, 8.0 mg/mL) were priorly prepared. Samples were mixed with 5 mL of dichloromethane/methanol (volume ratio: 2:1) solution for ultrasonication in ice water for 30 min with subsequent centrifugation under 10,000 rpm for 20 min to collect the extracts. The extraction process was repeated for 2 times. All the extracts were then stratified by adding 3 mL of 0.9% sodium chloride solution respectively, and centrifuged for 5 min at 4000 rpm. The organic solvent containing lipid was evaporated under nitrogen flow, followed by being redissolved with 0.2 ml acetone and 1.8 ml 1.5% sulfosalicylic acid. The absorbance of the mixture was measured at 440 nm for corelating the lipid concentration of sample with standard curve[6].

Using a carbon content of 0.53 g/g in protein of 0.76 g/g in lipid and of 0.40 g/g in carbohydrate[7], the intrinsic allocation percentage of carbon in carbohydrates, proteins and lipids of algae was determined.

S7. Detail parameters for HPLC and MS analysis

The LC analysis was performed on a Vanquish UHPLC System (Thermo Fisher Scientific, USA). Chromatography was carried out with an ACQUITY UPLC ® HSS T3 (2.1×100 mm, 1.8 µm) (Waters, Milford, MA, USA). The column maintained at 40 °C. The flow rate and injection volume were set at 0.3

mL/min and 2 μL, respectively. For LC-ESI (+)-MS analysis, the mobile phases consisted of (B2) 0.1% formic acid in acetonitrile (v/v) and (A2) 0.1% formic acid in water (v/v). Separation was conducted under the following gradient: 0~1 min, 8% B2; 1~8 min, 8%~98% B2; 8~10 min,98% B2; 10~10.1 min,98%~8% B2; 10.1~12 min, 8% B2. For LC-ESI (-)-MS analysis, the analytes were carried out with (B3) acetonitrile and (A3) ammonium formate (5mM). Separation was conducted under the following gradient: 0~1 min, 8% B3; 1~8 min, 8%~98% B3; 8~10 min, 98% B3; 10~10.1 min, 98%~8% B3; 10.1~12 min, 8% B3. Mass spectrometric detection of metabolites was performed on Orbitrap Exploris 120 (Thermo Fisher Scientific, USA) with ESI ion source. Simultaneous MS1 and MS/MS (Full MS-ddMS2 mode, data-dependent MS/MS) acquisition was used. The parameters were as follows: sheath gas pressure, 40 arb; aux gas flow, 10 arb; spray voltage, 3.50 kV and -2.50 kV for ESI(+) and ESI(-), respectively; capillary temperature, 325 ℃; MS1 range, m/z 50-1000; MS1 resolving power, 60000 FWHM; number of data dependent scans per cycle, 4; MS/MS resolving power, 15000 FWHM; normalized collision energy, 30%; dynamic exclusion time, automatic.

For C-14 substitution identification, the raw MS data files (.raw) were converted to .mzXML (for full scan mode) and .mgf (for ddMS2 mode) format using ProteoWizard (version 3.0.20360). The raw data files (.mzXML) were imported into El-MAVEN (version 0.12.0) for extraction of labeled metabolites[8]. In El-MAVEN, the labeled metabolite was defined if any isotopologues except M0 had the labeled fraction >0.02 in >50% of samples. The detailed parameters of current project in El-MAVEN were provided in Supplementary Table 1. Since there is no programs or tools specifically applicable for C-14 isotopic tracing, we applied the C-14 labeled MS data in the isotopic tracer mode for C-13 that provided by El-MAVEN, with simultaneous input of C-13 labeled MS data for alignment, annotation and comparison. C-13 incorporated samples were prepared same as that of C-14, via trophic transfer exposure.

Supplementary Figures

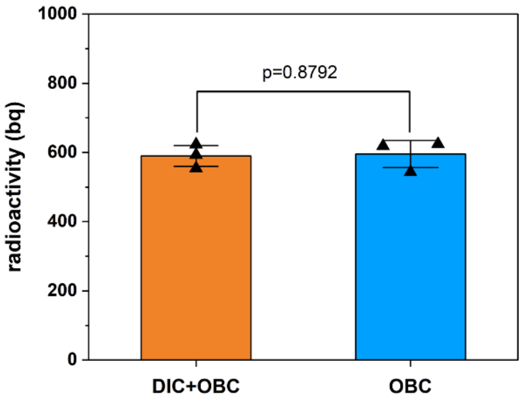

Supplementary Fig. 1. Comparison of the measured radioactivity in algae samples that being washed by 0.1 mM HCl to remove DIC or not. The acidified sample was adjusted to neutral pH before being subjected to LSC measurement.

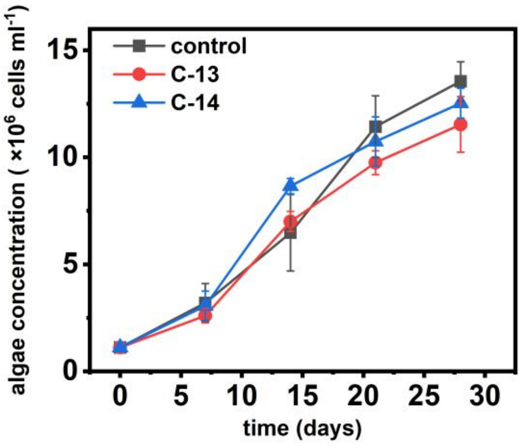

Supplementary Fig. 2. The growth curve of *D. salina* in 28 days cultivation. Algae was cultured in SE medium containing isotopic labeled $NaHCO_3$ (^{13}C- $NaHCO_3$ or ^{14}C- $NaHCO_3$) at a concentration of 0.172 mg l^{-1}.

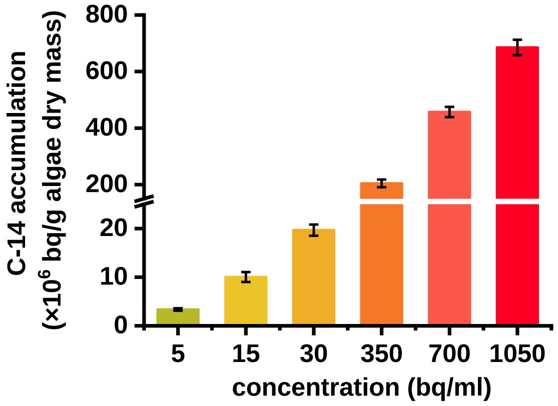

Supplementary Fig. 3. Accumulation of C-14 in *S. obliquus* under the cultivation in different medium containing various ^{14}C- $NaHCO_3$ at concentration of 5, 15, 30, 350, 700 and 1050 bq ml^{-1}, respectively.

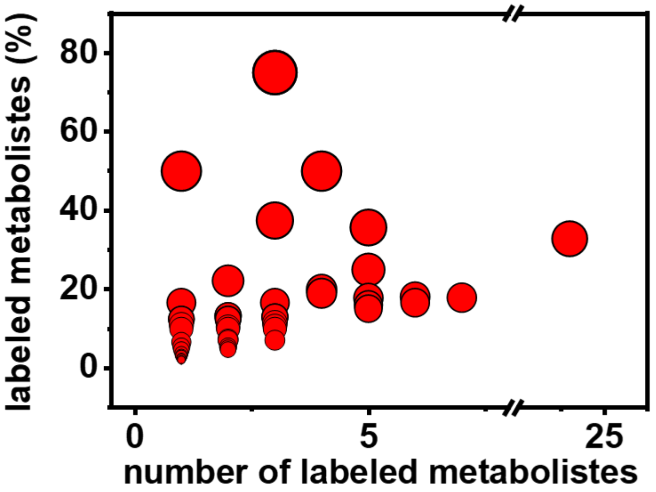

Supplementary Fig. 4. Distributions of 203 OBC-13 labeled metabolites from 52 metabolic pathways in zebrafish brain in the metabolic pathways. The circle size in the right panel represents the ratio of the number of labeled metabolites to the number of metabolites in a pathway.

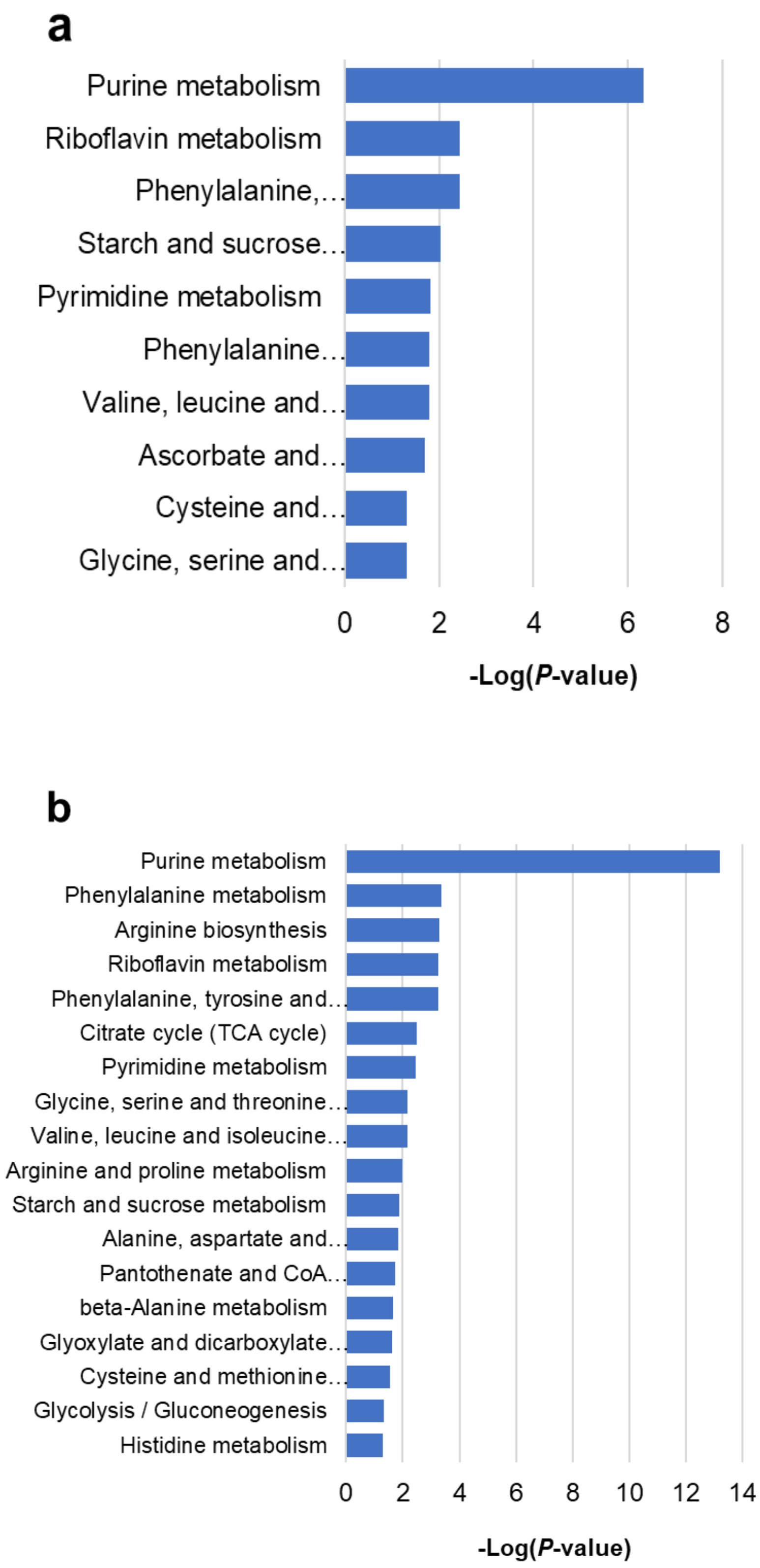

Supplementary Fig. 5. KEGG pathway analysis of (a) C-14 labeled or (b) C-13 labeled metabolites. The vertical axis represents the pathway category and the horizontal axis represents the enrichment score [−log(P-value)] of the pathway. Significantly enriched KEGG pathways ($P < 0.05$) are presented.

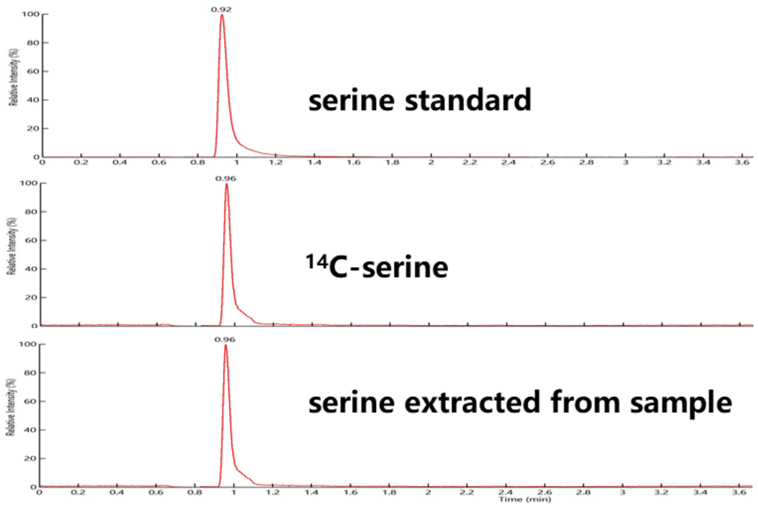

Supplementary Fig. 6. Retention time comparison of analytical standard of L-serine, ^{14}C-serine and serine extracted from fish brain.

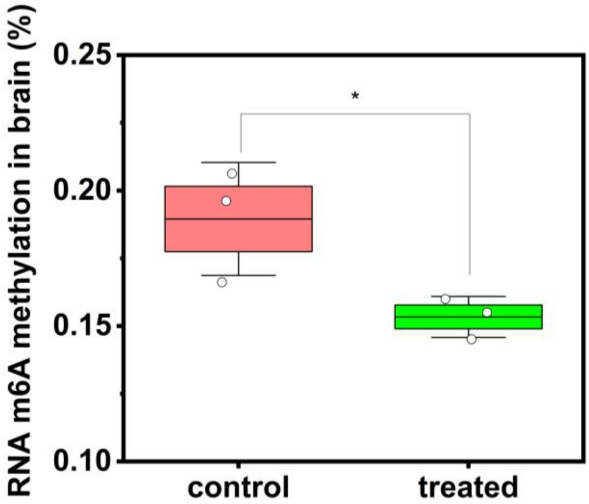

Supplementary Fig. 7. Alteration of RNA m6A methylation level after being exposed to C-14 via trophic transfer.

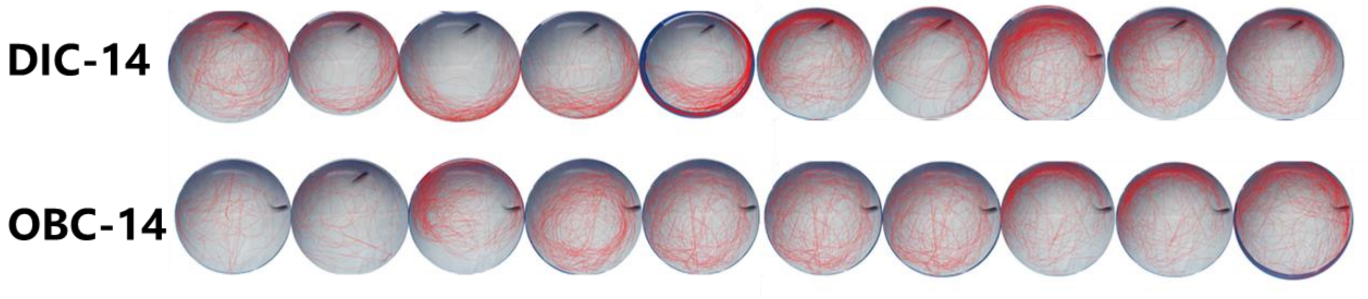

Supplementary Fig. 8. Trajectories recorded from zebrafish ingested OBC-14 via trophic transfer (with a fish body burden of 13.9 bq mg-1) or directly exposed to DIC-14 medium at a higher concentration of 1000 bq ml-1.

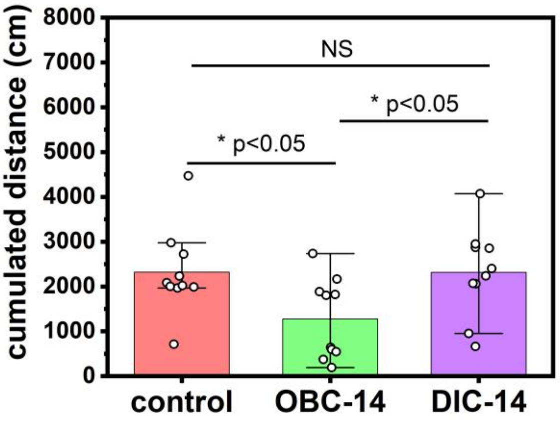

Supplementary Fig. 9. Cumulated swimming distance of zebrafish ingested OBC-14 via trophic transfer (with a fish body burden of 13.9 bq mg^{-1}) or directly exposed to DIC-14 medium at a higher concentration of 1000 bq ml^{-1}.

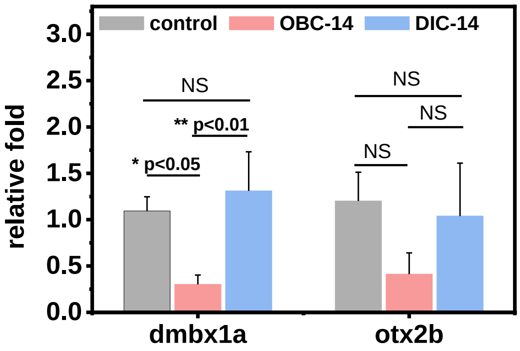

Supplementary Fig. 10. Relative RNA expression of *dmbx1a* and *otx2b* in zebrafish brain after being exposure to C-14 via trophic transfer or directly exposed to DIC-14 medium. Statistically significant differences were performed using Student-t tests, and asterisks (*) indicate statistically significant differences (*p < 0.05, **p < 0.01, ***p <0.001, and ****p < 0.0001).

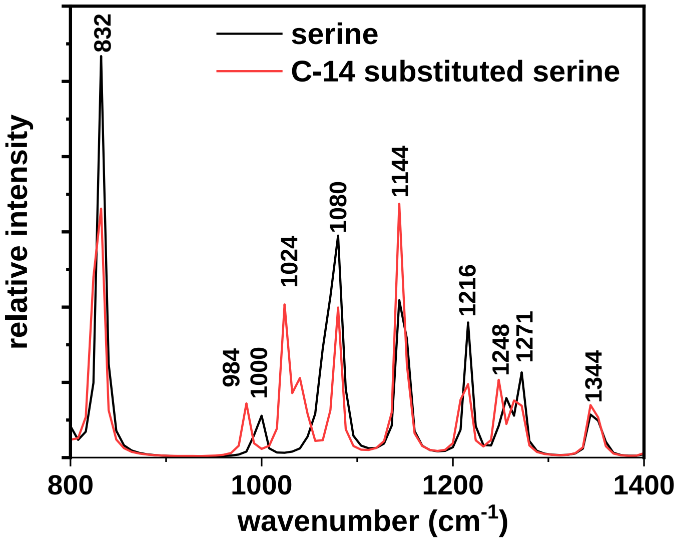

Supplementary Fig. 11. Vibrational frequency of serine or C-14 substituted serine at position 3 in serine where demethylation occurs, obtained by density functional theory (DFT) calculation (6-31G* basis set).

Supplementary Table 1. The parameters for El-MAVEN

**Routine**	Parameter
**Instrumentation**	Polarity = Auto Detect<br>Ionization Type = ESI
**Peak Detection**	EIC Smoothing Algorithm = Savitzky Golay<br>EIC Smoothing Window = 10 scans<br>Max Retention Time difference between Peaks = 0.25 min<br>Allow top [x] percent intensities as part of the real signal = 80 %<br>Baseline Smoothing = 5 scans

**Peak Filtering**	Min. Signal Baseline Difference = 3<br>Min. Peak Quality = 0.0<br>Isotope Peak Filtering is same as Peak Filtering: check
**Isotope settings**	Report Isotopes: check<br>Isotopic tracer: C13<br>Minimum signal correlation with parent isotopologues = 0.2%<br>Maximum RT deviation from parent isotopologues = 5 s<br>Link isotope peak RT range with parent peak: Check
**Alignment**	Maximum number of iterations = 10<br>Polynomial Degree = 5<br>At least [X] good peaks in group = 0<br>Limit total number of groups = 1000<br>Peak grouping window = 10<br>Minimum peak intensity = 300<br>Minimum peak S/N Ratio = 3<br>Minimum peak width = 5 scans
**Feature detection selection**	Compound database search : check<br>EIC Extraction window = 25<br>Match Retention Time : check 0.25 min<br>Limit number of reported groups per compound = 3 best
**Group filtering**	Consider overlap: check<br>distX weight = 1<br>distY weight = 1<br>overlap weight = 1

Supplementary Table 2. List of C-13 labeled metabolites and names of KEGG pathways

C-13 labeled metabolites	KEGG ID
1-Methylhistidine	C01152
DL-2-Aminooctanoic acid	C02261
3-Hydroxybutyryl-CoA	C03561
3-Phosphoglyceric acid	C00597
gamma-Aminobutyric acid	C00334
5-Methyltetrahydrofolic acid	C00440
AICA-riboside	C04663
N-alpha-Acetyl-L-lysine	C12989
Adenine	C00147
Adenosine	C00212

Adenosine phosphosulfate	C00224
ADP	C00008
ADP-glucose	C00498
Oxoglutaric acid	C00026
Aminoadipic acid	C00956
Adenosine monophosphate	C00020
L-Arginine	C00062
Ascorbic acid	C00072
L-Aspartic acid	C00049
Adenosine triphosphate	C00002
Vanillylmandelic acid	C05584
Betaine	C00719
L-Carnitine	C00487
CDP-ethanolamine	C00570
Cholesterol sulfate	C18043
Citraconic acid	C02226
Citric acid	C00158
Creatine	C00300
Creatinine	C00791
L-Cystathionine	C02291
Cytidine	C00475
Deoxyadenosine	C00559
Deoxyguanosine	C00330
Deoxyinosine	C05512
dGDP	C00361
2'-Deoxyguanosine 5'-monophosphate	C00362
dGTP	C00286
Dimethylglycine	C01026
Pipecolic acid	C00408
FAD	C00016
Flavone	C15608
Flavin mononucleotide	C00061
Fructose 1,6-bisphosphate	C00354
Fructose 6-phosphate	C00085
Glycerol	C00116
Guanosine diphosphate	C00035
Glucose 1-phosphate	C00103
Glucose 6-phosphate	C00092
Glutamic acid	C00302
Glutamine	C00064
Oxidized glutathione	C00127
Guanosine monophosphate	C00144

Guanosine triphosphate	C00044
Guanine	C00242
Guanosine	C00387
Histidine	C00135
L-Homoserine	C00263
ortho-Hydroxyphenylacetic acid	C05852
4-Hydroxyproline	C01157
Hypoxanthine	C00262
Indoleacrylic acid	C00954
Inosine	C00294
Isocitric acid	C00311
Leucine	C00123
(R)-Lipoic acid	C16241
Lysine	C00047
Malic acid	C00149
Malonyl-CoA	C00083
Methionine	C00073
N-Acetylglutamine	K11067
NADP	C00006
Asymmetric dimethylarginine	C03626
Niacinamide	C00153
O-Acetylserine	C00979
Ornithine	C00077
Pantothenic acid	C00864
Phenylalanine	C00079
Phenyllactic acid	C01479
Phenylpropiolic acid	C05629
Phenylpyruvic acid	C00166
Phosphoenolpyruvic acid	C00074
Proline	C00148
Pyroglutamic acid	C01879
Riboflavin	C00255
Ribose 1-phosphate	C00620
S-Adenosylhomocysteine	C00021
Serine	C00065
5'-Methylthioadenosine	C00170
Glycerol 3-phosphate	C00093
Taurine	C00245
Taurodeoxycholic acid	C05463
L-Threonine	C00188
Thymidine	C00214
L-Tryptophan	C00078

L-Tyrosine	C00082
Uridine 5'-diphosphate	C00015
Uridine diphosphate glucose	C00029
Uridine diphosphate glucuronic acid	C00167
Uridine diphosphate-N-acetylglucosamine	C00043
Uridine 5'-monophosphate	C00105
Uracil	C00106
Uridine	C00299
L-Valine	C00183
Xanthine	C00385
Xanthosine	C01762

Supplementary Table 3. List of C-14 labeled metabolites

C-14 labeled metabolites	KEGG ID
N-alpha-Acetyl-L-lysine	C12989
Adenosine phosphosulfate	C00224
ADP	C00008
Adenosine triphosphate	C00002
Betaine	C00719
CDP-ethanolamine	C00570
Cholesterol sulfate	C18043
Creatine	C00300
dGDP	C00361
dGTP	C00286
dUMP	C00365
FAD	C00016
Flavin mononucleotide	C00061
Fructose 6-phosphate	C00085
Guanosine diphosphate	C00035
Glucose 1-phosphate	C00103
Glucose 6-phosphate	C00092
Guanosine triphosphate	C00044
Guanosine	C00387
Histidine	C00135

4-Hydroxyproline	C01157
Hypoxanthine	C00262
Inosine	C00294
Leucine	C00123
Methionine	C00073
NADP	C00006
Niacinamide	C00153
Phenylalanine	C00079
Phosphoenolpyruvic acid	C00074
Proline	C00148
Pyroglutamic acid	C01879
Serine	C00065
5'-Methylthioadenosine	C00170
Taurodeoxycholic acid	C05463
L-Tryptophan	C00078
L-Tyrosine	C00082
Uridine 5'-diphosphate	C00015
Uridine diphosphate glucose	C00029
Uridine diphosphate glucuronic acid	C00167
Uridine diphosphate-N-acetylglucosamine	C00043
Uracil	C00106
Uridine	C00299
L-Valine	C00183
Xanthosine	C01762

Reference

1. Dong S, Wu Z, Wang M *et al.* Assessing comparable bioconcentration potentials for nanoparticles in aquatic organisms via combined utilization of machine learning and toxicokinetic models. *SmartMat.* 2022; **4**(3): e1155.

2. OECD. *Test No. 305: Bioaccumulation in Fish: Aqueous and Dietary Exposure.* 2012.

3. Bradford MM. A rapid and sensitive method for the quantitation of microgram quantities of protein utilizing the principle of protein-dye binding. *Anal Biochem*. 1976; **72**(1): 248-254.

4. DuBois M, Gilles KA, Hamilton JK *et al.* Colorimetric method for determination of sugars and related substances. *Anal Chem*. 1956; **28**(3): 350-356.

5. Fan W, Liu Y, Xu X *et al.* Effects of HCO^{3-} and CO_2 conversion rates on carbon assimilation strategies in marine microalgae: Implication by stable carbon isotope analysis of fatty acids. *Plant Physiol Biochem*. 2024; **209**: 108530.

6. Ren X, Wei C, Yan Q *et al.* Optimization of a novel lipid extraction process from microalgae. *Sci Rep*. 2021; **11**(1): 20221.

7. Geider R, La Roche J. Redfield revisited: variability of C:N:P in marine microalgae and its biochemical basis. *Eur J Phycol*. 2002; **37**(1): 1-17.

8. Agrawal S, Kumar S, Sehgal R *et al.* El-MAVEN: A Fast, Robust, and User-Friendly Mass Spectrometry Data Processing Engine for Metabolomics. In: D'Alessandro A (ed.) *High-Throughput Metabolomics: Methods and Protocols*. New York, NY: Springer New York; 2019. 301-321.
